# MiABI5-like7-*MiFT3* regulatory module controls floral transition induced by mepiquat chloride in evergreen perennial mango (*Mangifera indica* L.)

**DOI:** 10.1093/hr/uhaf336

**Published:** 2025-12-08

**Authors:** Wentian Xu, Bin Zheng, Hongxia Wu, Xiaolong He, Meng Gao, Kunliang Xie, Yanan Wang, Rulin Zhan, Yuyao Gao, Songbiao Wang, Xiaowei Ma

**Affiliations:** Key Laboratory of Tropical Fruit Biology, Ministry of Agriculture and Rural Affairs; Key Laboratory for Postharvest Physiology and Technology of Tropical Horticultural Products of Hainan Province, South Subtropical Crops Research Institute, Chinese Academy of Tropical Agricultural Sciences, Zhanjiang, Guangdong 524091, China; Key Laboratory of Tropical Fruit Biology, Ministry of Agriculture and Rural Affairs; Key Laboratory for Postharvest Physiology and Technology of Tropical Horticultural Products of Hainan Province, South Subtropical Crops Research Institute, Chinese Academy of Tropical Agricultural Sciences, Zhanjiang, Guangdong 524091, China; Key Laboratory of Tropical Fruit Biology, Ministry of Agriculture and Rural Affairs; Key Laboratory for Postharvest Physiology and Technology of Tropical Horticultural Products of Hainan Province, South Subtropical Crops Research Institute, Chinese Academy of Tropical Agricultural Sciences, Zhanjiang, Guangdong 524091, China; Key Laboratory of Tropical Fruit Biology, Ministry of Agriculture and Rural Affairs; Key Laboratory for Postharvest Physiology and Technology of Tropical Horticultural Products of Hainan Province, South Subtropical Crops Research Institute, Chinese Academy of Tropical Agricultural Sciences, Zhanjiang, Guangdong 524091, China; Key Laboratory of Tropical Fruit Biology, Ministry of Agriculture and Rural Affairs; Key Laboratory for Postharvest Physiology and Technology of Tropical Horticultural Products of Hainan Province, South Subtropical Crops Research Institute, Chinese Academy of Tropical Agricultural Sciences, Zhanjiang, Guangdong 524091, China; Key Laboratory of Tropical Fruit Biology, Ministry of Agriculture and Rural Affairs; Key Laboratory for Postharvest Physiology and Technology of Tropical Horticultural Products of Hainan Province, South Subtropical Crops Research Institute, Chinese Academy of Tropical Agricultural Sciences, Zhanjiang, Guangdong 524091, China; Key Laboratory of Tropical Fruit Biology, Ministry of Agriculture and Rural Affairs; Key Laboratory for Postharvest Physiology and Technology of Tropical Horticultural Products of Hainan Province, South Subtropical Crops Research Institute, Chinese Academy of Tropical Agricultural Sciences, Zhanjiang, Guangdong 524091, China; Sanya Research Institute, Chinese Academy of Tropical Agricultural Sciences, Sanya, Hainan 572024, China; Key Laboratory of Tropical Fruit Biology, Ministry of Agriculture and Rural Affairs; Key Laboratory for Postharvest Physiology and Technology of Tropical Horticultural Products of Hainan Province, South Subtropical Crops Research Institute, Chinese Academy of Tropical Agricultural Sciences, Zhanjiang, Guangdong 524091, China; Key Laboratory of Tropical Fruit Biology, Ministry of Agriculture and Rural Affairs; Key Laboratory for Postharvest Physiology and Technology of Tropical Horticultural Products of Hainan Province, South Subtropical Crops Research Institute, Chinese Academy of Tropical Agricultural Sciences, Zhanjiang, Guangdong 524091, China; Sanya Research Institute, Chinese Academy of Tropical Agricultural Sciences, Sanya, Hainan 572024, China; Key Laboratory of Tropical Fruit Biology, Ministry of Agriculture and Rural Affairs; Key Laboratory for Postharvest Physiology and Technology of Tropical Horticultural Products of Hainan Province, South Subtropical Crops Research Institute, Chinese Academy of Tropical Agricultural Sciences, Zhanjiang, Guangdong 524091, China

## Abstract

Regulating floral induction (FI) through the application of gibberellin (GA) biosynthesis inhibitors is a critical agricultural practice to prevent yield loss in fruit trees. We observed that mepiquat chloride (MC), a highly safe plant growth retardant, enhanced FI in mango. Nevertheless, the molecular mechanism by which MC facilitates FI remains elusive. Using two distinct treatments and varied stages during FI in mango (*Mangifera indica* L. ‘Tainong No.1’), 24 dynamic transcriptome profiles were constructed. Through pairwise comparisons and weighted gene co-expression network analysis (WGCNA), a regulatory network centered on the hub gene *FLOWERING LOCUS T3* (*MiFT3*) was established. We further discovered MC-induced floral transition was associated with the decreases of GA_20_ and GA_3_ levels and the upregulation of *MiGA2oxs* (*GA2 OXIDASES*) expression, alongside the increase of abscisic acid (ABA) content and the upregulation of *MiNCED1* (*9-cis-epoxycarotenoid dioxygenase 1*) and *MiABI5-like7* (*ABSCISIC ACID-INSENSITIVE 5-like7*). Furthermore, biochemical assays and stable transgenic experiments were applied to confirmed that MiABI5-like7 activated the expression of *MiFT3*. Moreover, silencing *MiABI5-like7* in mango buds delayed floral transition, while ectopic expression of *MiABI5-like7* promoted early flowering. Additionally, exogenous ABA accelerated the floral transition induced by MC, whereas an ABA inhibitor delayed floral transition, which were associated with the expression levels of *MiABI5-like7* and *MiFT3*. This study clarified the mechanism by which MC induced floral transition by inhibiting GA biosynthesis that activate MiABI5-like7-mediated signaling pathway, which provides novel insights into the regulatory network of FI in plants and offers a solution for solving the issue of insufficient flowering in warm winter climates.

## Introduction

Mango is a globally cultivated fruit crop renowned for its appealing flavor and high nutritional value, predominantly grown in tropical and subtropical regions. The yield and quality of mango are significantly influenced by the density of flowering, which is initially determined by FI. Low temperatures play a crucial role in affecting mango flowering [1]. However, the phenomenon of global warming has resulted in warmer winter climates, often leading to reduced and unstable flowering rates in mango trees, thereby posing substantial challenges to mango production [2]. Unlike model plants, such as *Arabidopsis* and other herbaceous, mango FI is facilitated by reduced GA levels [3–5]. Using GA biosynthesis inhibitors like paclobutrazol (PBZ) helps counteract poor environmental conditions, such as inadequate cool temperatures, by reducing vegetative growth and promoting flowering [1, 5]. Although PBZ has been economically beneficial in mango-producing regions, its overuse has led to soil residue, tree aging, and inconsistent flowering [6, 7]. Therefore, the development of low-residual floral inducers and a comprehensive understanding of the regulatory mechanisms underlying FI are crucial for addressing the issue of insufficient flowering in warm regions.

Unlike temperate deciduous trees, the process of FI, initiation and development in most evergreen subtropical or tropical trees, is continuous and not interrupted by dormancy [1]. As an initial step of flowering process in trees, FI represents the transforming of vegetative into reproductive growth and is accompanied by structural and cytological changes in the buds, which is tightly controlled by environmental stimuli and intrinsic signals [8]. In *Arabidopsis thaliana*, flowering pathways, including vernalization, photoperiod, autonomous, GA, thermosensory, and aging pathways, have been proposed to integrate both environmental and developmental signals and converge to regulate various floral integrators, including *FLOWERING LOCUS T* (*FT*), *SUPPRESSOR OF OVEREXPRESSION OF CONSTANS 1* (*SOC1*), which determine the floral evocation [9]. To date, several flowering-related genes have been isolated and characterized in mango, such as *MiFTs*, *RING zinc finger protein* (*MiRZFP34*), a*nd CONSTANS-like 2 s* (*MiCOL2s*) [10, 11]. Notably, five *MiFTs* (*MiFT1a*, *MiFT1b*, *MiFT2*, *MiFT3*, and *MiFT4*) have been identified within the ‘SiJiMi’ mango genome, all of which have been shown to significantly enhance early flowering in *Arabidopsis* [10]. Among these, *MiFT1a*, *MiFT1b*, and *MiFT2* exhibited a greater efficacy in promoting early flowering, indicating that the FT gene family members possess both conserved and divergent functions in regulating flowering across plant species. Additionally, certain proteins that influence plant flowering have been found to interact directly with MiFTs. For example, MiRZFP34 has been identified to interact with MiFT2, revealing the intricate regulatory network governing mango flowering [11]. However, the molecular mechanisms governing FI in evergreen trees are still not as well understood as those in model plants and deciduous trees.

GA plays a pivotal role in the regulation of flowering in plants. In the GA biosynthesis pathway, activating enzymes like GA20-oxidase (GA20ox) and GA3-oxidase (GA3ox) convert GA_12_ into active GAs, while GA2-oxidases (GA2oxs) manage GA turnover to maintain proper GA levels [4]. In long-day herbaceous plants, the exogenous application of GAs can induce flowering under non-inductive conditions. Treatments with GA synthesis inhibitors like PBZ, overexpression of *GA2ox*, or loss of *GA20ox* can result in delayed flowering [12–14]. Conversely, in various perennial tree species, including grapevine and apple, GAs inhibit flowering, while PBZ application promotes it [15–17]. The disruption of the balance between GAs and other phytohormones through GA applications repressed flowering by up-regulating the floral repressor *TERMINAL FLOWER1* (*TFL1*) and down-regulating the floral integrators, such as *FT*, *SOC1* [18–20]. The growth repressors DELLA proteins (DELLAs) is known as one of the major components of GA signaling. DELLAs mediate transcription by sequestering flowering activators, including WRKY12 and SQUAMOSA promoter-binding protein-likes (SPLs), or by enhancing the flowering repressors, such as WRKY13, thereby suppressing the expression of the flowering time genes to inhibit flowering in *Arabidopsis* [9]. Numerous studies have explored the transcriptomes of buds during FI following GA treatments in woody fruit trees, with the objective of identifying potential flowering genes and GA-responsive transcription factors (TFs), such as DELLA, WRKY, and SPL [18, 20]. Compared to model plants, the regulatory modules that respond to GA signal to control FI in perennial fruit trees have not yet been well elucidated.

ABA is another critical phytohormone involved in FI and acts antagonistically to GAs in various physiological processes, including seed germination [21]. In certain woody species, ABA positively influences the regulation of flowering time. The application of ABA had been observed to down-regulate the expression of the *TFL1*, thereby promoting flower bud formation in apple [22]. Additionally, ABA up-regulated the expression of the floral meristem identity gene *APETALA1* (*AP1*), advancing flowering in litchi [23]. Nonetheless, the role of ABA in the floral transition of herbaceous plants remains a subject of debate. ABA delays floral transition by activating the floral repressor *FLOWERING LOCUS C* (*FLC*) through ABA signaling components ABSCISIC ACID INSENSITIVE 4 (ABI4) and (ABI5) in *Arabidopsis* [24]. ABA could also accelerate the floral transition of *Arabidopsis* through the photoperiodic response gene so as to respond to drought escape [25]. The different roles of ABA in floral transition might rely on environmental conditions and the species-specific regulatory mechanism involving the crosstalk of ABA and flowering pathways [26]. To date, it remains unclear whether ABA participates in the GA-mediated regulation of flowering time in plants.

In comparison to PBZ, MC is extensively utilized as an environmentally friendly, low-residue, and safer plant growth regulator for crops, such as cotton and soybeans globally, significantly influencing plant growth, yield, and drought stress tolerance [27, 28]. MC was proposed to inhibit enzyme activity acting in the early stages of GA biosynthesis [29]. Exogenous MC modulated GA biosynthesis and signal transduction to control internode elongation in cotton [30], and it also facilitated lateral root formation by coordinating the metabolism and signal transduction of GA and ABA [31]. However, there is a paucity of research on the application of MC in perennial fruit crops, particularly concerning its role and regulatory mechanisms in FI, which necessitates further elucidation.

In this study, we verified that the application of MC significantly enhanced the process and efficiency of FI in ‘Tainong No. 1’ mango. Histological and RNA-seq analyses were employed to examine the cytological and transcriptomic alterations at various developmental stages of two treatments. Through WGCNA, core TFs and hub gene associated with floral transition were identified. By integrating changes in hormone levels with transcriptomic data, it was determined that the GA- and ABA-related pathways were closely linked to the FI treated with MC. Notably, we demonstrated that the ABA signal transcription factor MiABI5-like7 positively regulates the MC-induced floral transition by activating the expression of *MiFT3*, as evidenced. Ultimately, our findings suggested that the MC-induced floral transition in mango was dependent upon ABA signaling pathway.

## Results

### The flowering phenology of mango and the morphological development of terminal buds during FI

**Figure 1:**
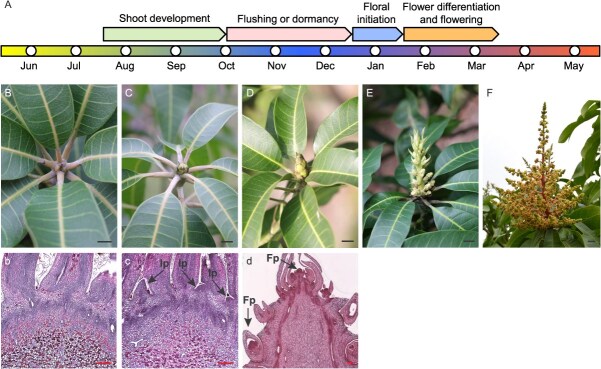
The flowering phenology, morphology and cytology of bud development in *Mangifera indica* L. (A) Diagram displaying the complete cycle of mango trees from vegetative to flowering, clarifying the floral transition in late December to early January of the next year, floral differentiation and flowering in mid-January to early March. (B–F) Visual display of a bud changing into a panicle. (b–d) Parafffn section observations of a vegetative bud changing into an initial-floral bud. (B, b) vegetative bud. (C, c) green-tip bud with the occurrence of inflorescence primordium (Ip, arrows). (D, d) initial-floral bud with floret primordium (Fp, arrows), presenting a typical inflorescence structure. (E) Panicle at the elongated stage. (F) Panicle at the anthesis. Scale bars: (B–E) 0.5 cm; (F) 1 cm; (b–d) 200 μm.

FI is a critical aspect of mango cultivation in the coastal production areas of South China. Following the harvest of fruit, mango trees undergo pruning, which typically occurs from mid to late July. This is succeeded by a period of new shoot development from late July to late September. In the absence of external interventions, the mango tree may experience multiple cycles of shoot development; most shoots (over 75%) (Fig. S1a) will initiate a new cycle of growth, commonly referred to as ‘flushing’, while others will cease growth and enter a dormant phase (Fig. 1A). During this dormancy, the terminal buds of the most recent shoots remain small and are tightly encased by bracts, referred to as vegetative buds (Fig. 1B). Between late December and early January of the subsequent year, the bracts of certain dormant buds begin to loosen, exposing green tips known as green-tip buds (Fig. 1C). Following mid to late January, these green-tip buds develop into initial-floral buds, characterized by bracts that are on the verge of abscission, commonly referred to as ‘brush pen heads’ (Fig. 1D). Subsequently, these initial-floral buds progress into the stages of flower differentiation and flowering (Fig. 1E and F). Different from other temperate deciduous fruit trees and some herbaceous flower plants, the floral initiation and floral development in mango is a continuous process without interruption of dormancy (Fig. 1A).

Histological analyses reveal that the meristematic region of the vegetative bud exhibited a dome-shaped morphology (Fig. 1b), whereas the green-tip bud displayed pronounced swelling and lateral expansion, presenting the inflorescence primordium in the lateral meristematic zone (Fig. 1c). Additionally, the initial-floral bud was observed to possess floret primordium, presenting a typical inflorescence structure (Fig. 1d). According to the statistics for three consecutive years (2020–2022), the ratio of floral bud formation is often less than 30% (Fig. S1b) without external control in mango.

### Exogenous MC application promotes the process and efficiency of FI

**Figure 2:**
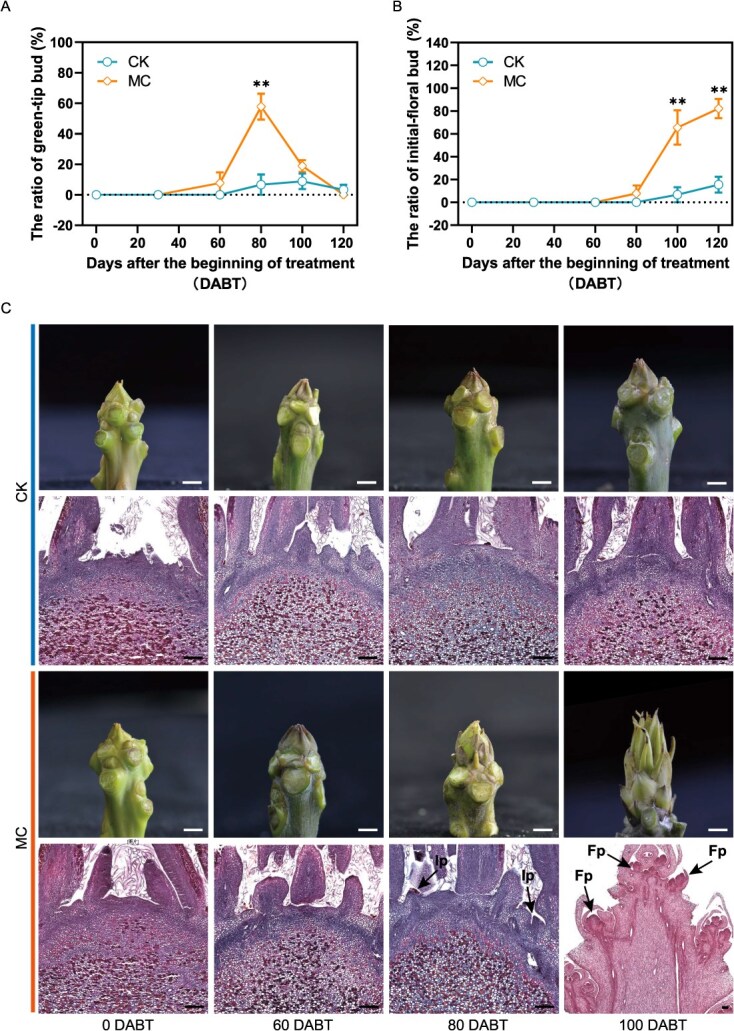
Exogenous MC application can promote the process and efficiency of FI. (A) The green-tip ratio during different stages of FI. (B) The initial-floral bud ratio during different stages of FI. Values are mean ± standard error. ^**^P < 0.01 (*n* = 3, multiple *t*-tests). (C) The phenotypic and cytological changes of bud during FI stage (0, 60, 80, and 100 DABT) following MC treatment and water spray (CK). DABT, different days after the beginning of treatment; MC, 2 g·l^−1^ treatment plants; CK, water-spraying plants; Ip, inflorescence primordium; Fp, floret primordium. Scale bars: white 0.5 cm; black 200 μm.

In the preliminary investigation, we conformed that a concentration of 2 g·l^−1^ MC had a significant impact on floral bud ratio and yield in mango (Fig. S2). To understand how MC affected the process and efficiency of FI, the ratios of green-tip bud and initial-floral bud from the control (CK) and MC-treated plants were evaluated at different days after the beginning of treatment (DABT) (Fig. 2A and B). Prior to 60 DABT, no green-tip or initial-floral buds were observed, with vegetative buds prevailing in both MC-treated and control plants. At 60 DABT, a minor emergence of green-tip buds (less than 10%) was noted exclusively in MC-treated plants, while control plants exhibited no such development. By 80 DABT, the green-tip bud ratio peaked at 57.8%, and an initial floral bud ratio of 7.8% was observed in MC-treated plants. At 100 DABT, the initial-floral bud was predominant and its ratio reached 65.6% due to the transition from green tip to initial-floral bud in MC-treated plants, suggesting that these plants had largely completed FI and were entering the floral differentiation stage. In contrast, the ratios of green tip and initial-floral bud in the CK plants were lower than 10% at both 80 and 100 DABT, indicating that these plants were primarily in the vegetative stage. After 120 DABT, the green-tip bud ratio at or near 0 was exhibited, and the formation of flower bud had nearly stabilized in both MC-treated and control plants, with floral bud ratios of 82.2% and 15.6% observed in the MC-treated and control plants, respectively. Collectively, these results suggest that MC treatment enhances the process and efficiency of FI in ‘Tainong No. 1’ mango plants.

Additionally, cytological examinations of the buds at various intervals (0, 60, 80, and 100 DABT) following MC and CK treatments revealed no significant differences in the meristematic zone of the buds between the two treatments prior to 60 DABT (Fig. 2C). The buds treated with MC demonstrated the initiation of inflorescence primordium at 80 DABT, which further develops into a typical conical inflorescence structure at 100 DABT. Conversely, the apical buds in the control group remained in a vegetative state throughout the observation period. The morphological and statistical analyses indicate that the green-tip bud is a critical morphological marker of the floral transition, with its ratio influencing the final floral bud rate. Based on the green-tip bud ratio, the FI period was segmented into three phases: Phase E, the early phase of FI (0–60 DABT); Phase M, the middle phase of FI (60–80 DABT); and Phase L, the late phase of FI (80–100 DABT).

### Global transcriptome analysis of MC-treated buds during FI

To examine the transcriptional changes of mango FI in response to MC, we constructed 24 cDNA libraries for both MC-treated and control buds at 0, 60, 80, 100 DABT (represented by At, Bt, Ct, Dt). For convenience, treatment and control samples were referred to as TAt, TBt, TCt, TDt and At, Bt, Ct, Dt, respectively. The detailed statistics of RNA-seq data for each library were shown in Table S1. Overall, an average clean base of 7.04 Gb was generated, with >94% Q30 base percentage and a GC content of 42.28 to 43.53% in all libraries, and the percentage of clean reads uniquely mapped to the mango genome was >91.42%. In addition, principal component analysis (PCA) (Fig. S3a) of all samples presented distinct development stages both for MC-treatment and control samples, and Pearson's correlation coefficient within the biological replicates was >0.96 (Fig. S4), confirming high reliability of the sequencing data.

Subsequently, pairwise comparisons were carried out to obtain differentially expressed genes (DEGs) using thresholds of fold-change ≥2 and FDR < 0.05. For the successive developmental stages by MC treatment, 3149, 4901, and 3801 DEGs were identified in the TBt vs TAt, TCt vs TBt, and TDt vs TCt, respectively (Fig. S3b). For the MC-treated and control groups at the same time point, there were 975, 5486, and 5788 DEGs in the TBt vs Bt, TCt vs Ct, and TDt vs Dt, respectively (Fig. S3b). The results showed that exogenous MC influenced gene expression mostly at 80 and 100 DABT.

### MC-induced DEGs during different phases of FI

**Figure 3:**
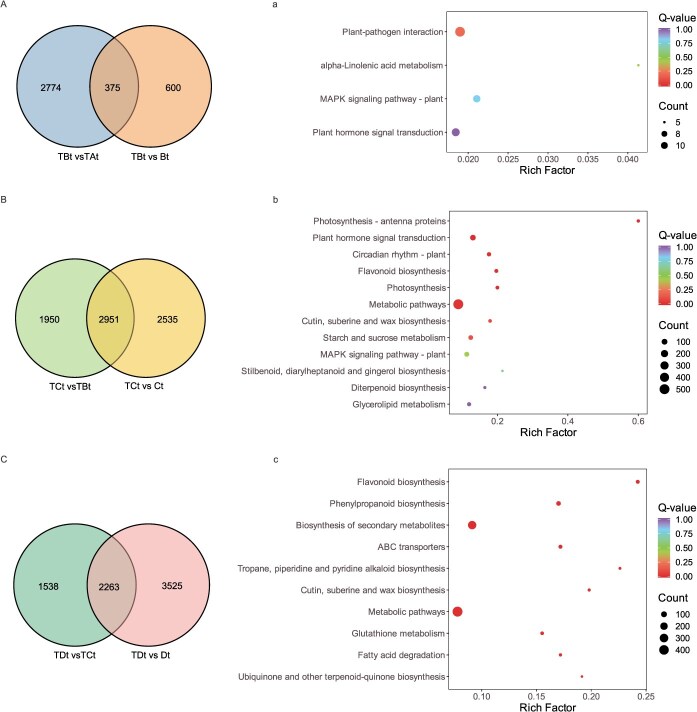
Analysis of DEGs in pairwise comparisons of different stages between MC-treated and water spray (CK) buds. (A) Venn diagram of DEGs among TBt vs TAt and TBt vs Bt. (B) Venn diagram of DEGs among TCt vs TBt and TCt vs Ct. (C) Venn diagram of DEGs among TDt vs TCt and TDt vs Dt. (a–c) KEGG enrichment analysis of the MC-induced DEGs at the early, middle, late phase of FI, respectively. TAt, TBt, TCt and TDt represent MC-treated samples at 0, 60, 80, 100 DABT, respectively. At, Bt, Ct, and Dt represent CK samples at 0, 60, 80, 100 DABT, respectively.

To find the MC-induced DEGs and their functionality during the course of FI, we performed Venn diagrams analysis among different pairwise comparisons and KEGG enrichment analysis for the shared DEGs in a certain phase in turn. Among TBt vs TAt and TBt vs Bt, 375 common DEGs were regarded as MC-induced DEGs at Phase E (Fig. 3A). These shared DEGs were enriched in the pathways including ‘MAPK signaling pathway-plant’, ‘Plant hormone signal transduction’, and ‘alpha-Linolenic acid metabolism’ (Fig. 3a), indicating that various stress responses were involved in Phase E. Similarly, among TCt vs TBt and TCt vs Ct, a total of 2951 common DEGs, considering as MC-induced DEGs at Phase M (Fig. 3B), were found to be enriched in the pathways related to ‘Plant hormone signal transduction’, ‘Circadian rhythm - plant’, ‘MAPK signaling pathway-plant’, and ‘Diterpenoid biosynthesis’ (Fig. 3b). These implied that responses of GA-related transcription and flowering timing mostly occurred in Phase M. Furthermore, 2263 shared DEGs among TDt vs TCt and TDt vs Dt were recognized as MC-induced DEGs at Phase L (Fig. 3C), and were mostly enriched in pathways ‘Flavonoid biosynthesis’, ‘Phenylpropanoid biosynthesis’, and ‘Biosynthesis of secondary metabolites’ (Fig. 3c), suggesting diverse plant metabolism responses in Phase L. After removing the genes that were duplicated in the above three shared gene sets (375, 2951, 2263), a total of 4847 DEGs were obtained and regarded as MC-induced DEGs during the course of FI, which were selected for further analysis.

### MC-induced specific DEGs and TFs at floral transition stage

**Figure 4:**
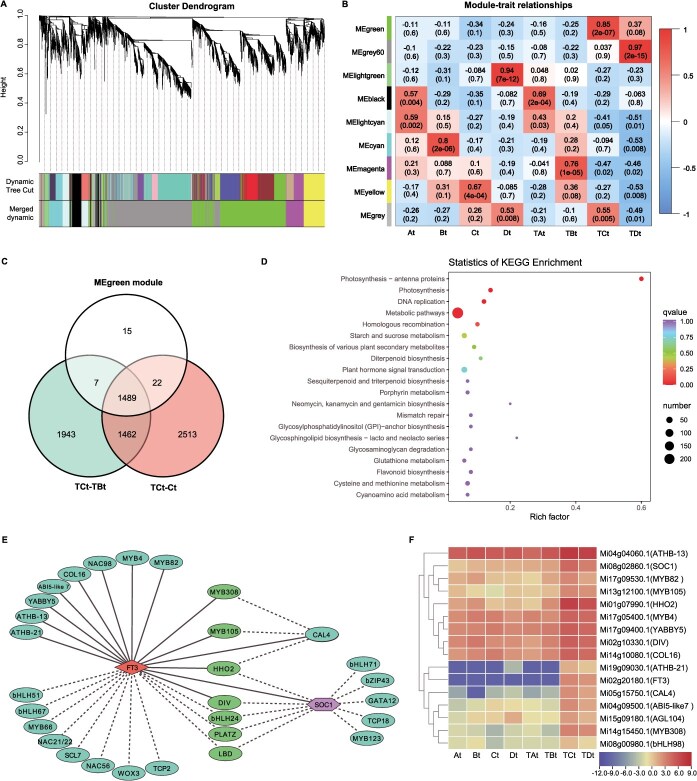
Regulatory network related to MC-induced floral transition and key genes. (A) Hierarchical cluster tree displaying co-expression modules identified by WGCNA. The tree branches comprise nine modules marked by different colours. (B) Correlations between module and sample. Each row represents a module. Each column represents a specific sample. The number at the row–column intersection represents the correlation coefficient and the *P* value between the module and sample. (C) Venn diagram of MC-induced specific DEGs at floral transition stage. (D) KEGG enrichment analysis of the MC-induced specific DEGs at floral transition stage. (E) Co-expression network analysis of three flowering time related genes (*MiFT3*, *MiSOC1*, and *MiCAL4*) and TFs in the MEgreen module. The weighted Pearson correlation coefficient of each gene pair exceeding 0.33 is represented by a solid line, otherwise it is represented by a dashed line. (F) The heat map displays the relative FPKM of three flowering time related genes (*MiFT3*, *MiSOC1*, and *MiCAL4*) and 13 core TFs within module MEgreen.

To further explore MC-induced specific genes highly correlated with certain phases of FI, we employed WGCNA to analyze the co-expressed relationship among 4847 DEGs during FI. A total of nine distinct modules labeled by variant colors were produced (Fig. 4A and B, Table S2), and gene set in MEmagenta, MEgreen and MEgrey60 modules was highly correlated with TBt, TCt, and TDt samples, with correlation coefficients of 0.76 (*e*-value = 1 × 10^−5^), 0.85 (*e*-value = 2 × 10^−7^), 0.97 (*e*-value = 2 × 10^−15^), respectively (Fig. 4B).

The MEgreen module contained a total of 1533 genes, with 1489 genes belonging to 2951 common DEGs among TCt vs TBt and TCt vs Ct (Fig. 4C, Table S3). According to the morphological and statistical results (Fig. 2), the green-tip bud was predominant at TCt tissues, which comprised the floral transition stage. The 1489 genes (86 encoding TFs) in the MEgreen module were mainly highly expressed at TCt tissue and regarded as specific DEGs of MC response in Phase M (Table S3). There were 1489 genes in the MEgreen module that were assigned to the KEGG pathway ‘Plant hormone signal transduction’, ‘Diterpenoid biosynthesis’, ‘Circadian rhythm-plant’ (Fig. 4D). For genes in the Circadian rhythm–plant pathway, we identified a transcript of *MiFT* (*Mi02g20180.1*) sharing 99% homology with the previously reported mango flowering-related gene *MiFT3* (JQ700254) [32], which had the highest expression level at the TCt tissues in this study, implying a vital flowering signal specific to green-tip bud (Table S3).

Furthermore, 86 TFs highly expressed during at TCt tissue were classified and mainly belonged to the *MYB*, *bHLH*, *NAC*, *AP2/ERF*, *MADS-box*, *GRAS*, *bZIP* (Table S4), indicating that these TFs may play a vital role in the regulation of floral transition in response to MC in mango. Interestingly, three MADS-box TFs related to flowering time were also identified among the 86 TFs, including *SOC1* (*Mi08g02860.1*, GO:0009908), *CAULIFLOWER 4* (*CAL4*, *Mi05g15750.1*, GO:0009908), and *AGAMOUS-Like 104* (*AGL104*, Mi15g09180.1, GO: 0010152), and their expression pattern in the course of FI induced by MC is similar to that of the *MiFT3* (Table S4).

### Co-expression network and core regulators related to MC-induced floral transition

To further reveal the relationship between flowering time related genes and TFs in Phase M, the co-expressed networks of candidate genes and TFs in MEgreen module were constructed based on the edge weights and visualized using Cytoscape 3.9.1, respectively. In the MEgreen module, a network including three flowering time related genes (one *MiFT3*, one *MiSOC1*, and one *MiCAL4*) were obtained, of which the *MiFT3* presented the most connections with other peripheral genes, suggesting that this gene was more critical and considered hub gene in the network (Fig. 4E). The weight value indicates the connection strength of each gene pair in the module, and a higher weight value means that the gene pair has stronger relationship. Gene pairs with a high weight value (weight > 0.33) were indicated by solid lines in this network. Interestingly, thirteen TFs had stronger relationship with the *MiFT3*, including six *MYBs*, two *HD-ZIP* (*ATHB-13* and *ATHB-21*), one *COL16* (*CONSTANS-like16*), one *ABI5-like7* (*ABSCISIC ACID-INSENSITIVE 5-like7*) related to ABA-activated signaling pathway (GO: 0009738), one *CAL4* related to flower development (GO: 0009908), one *YABBY5* related to regulation of shoot apical meristem development (GO: 1902183) and one *NAC98* related to primary shoot apical meristem specification (GO: 0010072). The expression levels of these TFs in the network were closely related to MC-induced floral transition (Fig. 4F), and they were most likely to be the potential governors of expression of *MiFT3* in mango.

In addition, nine genes associated with flowering and hormone pathways were selected for reverse transcription quantitative real-time polymerase chain reaction (RT-qPCR) analysis (Fig. S5). The findings revealed a significant correlation between the transcriptomic and RT-qPCR data (*R*^2^ = 0.81), thereby affirming the reliability of the transcriptome data.

### GA and ABA-related pathways are strongly associated with MC-induced floral transition

**Figure 5:**
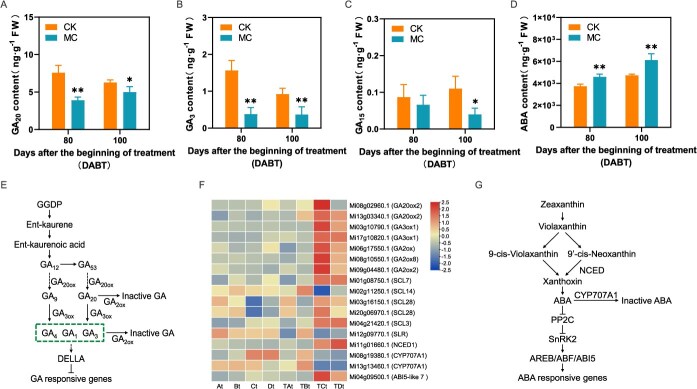
Overview of major GAs, ABA and their metabolism and signaling pathways following MC and water treatment (CK). (A–D) Levels of major GAs and ABA at 80 and 100 DABT in MC-treated and the water spray (CK) groups. Values are mean ± standard error. ^**^P < 0.01 ^*^P < 0.05 (*n* = 3, Student's *t*-test). (E) GA biosynthesis and signal transduction. (F) Heat map displaying the expression levels of DEGs related to GA and ABA synthesis and signal transduction at different stages of FI in response to MC. (G) ABA biosynthesis and signal transduction.

Given the strong association of floral transition with the ‘Plant hormone signal transduction’ and ‘Diterpenoid biosynthesis’ pathways, we investigated the contents of GAs in buds at 80 and 100 DABT, as well as the expression changes of the DEGs related to GA synthesis and signal transduction between control and MC-treated groups. Among the ten GAs measured, three (GA_20_, GA_3_ and GA_15_) were detected across all samples, whereas seven (GA_1_, GA_4_, GA_7_, GA_9_, GA_19_, GA_24_ and GA_53_) were not detected in any samples (Table S5). At 80 DABT, the levels of GA_20_ and GA_3_ were significantly reduced in MC-treated buds (Fig. 5A and B), while no significant differences in GA_15_ levels was observed between the control and MC-treated buds (Fig. 5C), indicating that GA_20_ and GA_3_ may play a crucial role in floral transition following MC application.

In the GA biosynthesis pathway, the expression of seven GA metabolism genes was induced by MC at 80 DABT (Fig. 5E and F). In MC-treated buds, two *GA20ox*s and two *GA3oxs* were significantly up-regulated at 80 DABT, suggesting a feedback regulation mechanism of GA biosynthesis in response to exogenous MC. We identified three *GA2oxs* whose expression was significantly up-regulated at 80 DABT in MC-treated buds, indicating that the enhanced expression of GA catabolism genes, specifically GA2oxs, may be the primary factor contributing to the suppression of GA biosynthesis by MC. DELLA proteins, as pivotal regulators in the GA signaling transduction pathway, play a role in modulating both downstream GA responses and upstream GA homeostasis. Here, we identified six genes encoding DELLA proteins, including five *MiSCLs* (*SCARECROW-LIKE*) and one *MiSLR* (*SLENDER RICE-LIKE*). Among these, four genes exhibited significant upregulation, while two showed significant downregulation at 80 DABT in response to exogenous MC application (Fig. 5E and F). Collectively, the results indicate that the application of MC led to a reduction in GA_20_ and GA_3_ contents by modulating the expression of genes associated with GA biosynthesis and signal transduction.

In addition to GA, ABA also plays a major role in the induction of flowering [9]. In this study, MC treatment significantly elevated endogenous ABA levels in buds between 80 and 100 DABT (Fig. 5D). Within the ABA biosynthetic pathway, the expression level of *MiNCED1* (*9-cis-epoxycarotenoid dioxygenase 1*) was significantly up-regulated in MC-treated buds during the same period, whereas the expression of two genes encoding *ABA 8*′*-hydroxylase 1* (*MiCYP707A1*) was significantly down-regulated at 80 and 100 DABT (Fig. 5F and G), suggesting that the increased expression of ABA biosynthetic gene *MiNCED1*, coupled with the decreased expression of the ABA degradation genes *MiCYP707A1s* contributed to the elevated ABA levels. Additionally, the ABA signaling component *MiABI5-like7* (*Mi04g09500.1*) within module MEgreen was significantly up-regulated in MC-treated buds at 80 DABT, potentially serving as a regulator of *MiFT3* expression in the aforementioned analysis (Fig. 5F and G).

### MiABI5-like7 positively regulates floral transition by activating the expression of *MiFT3*

**Figure 6:**
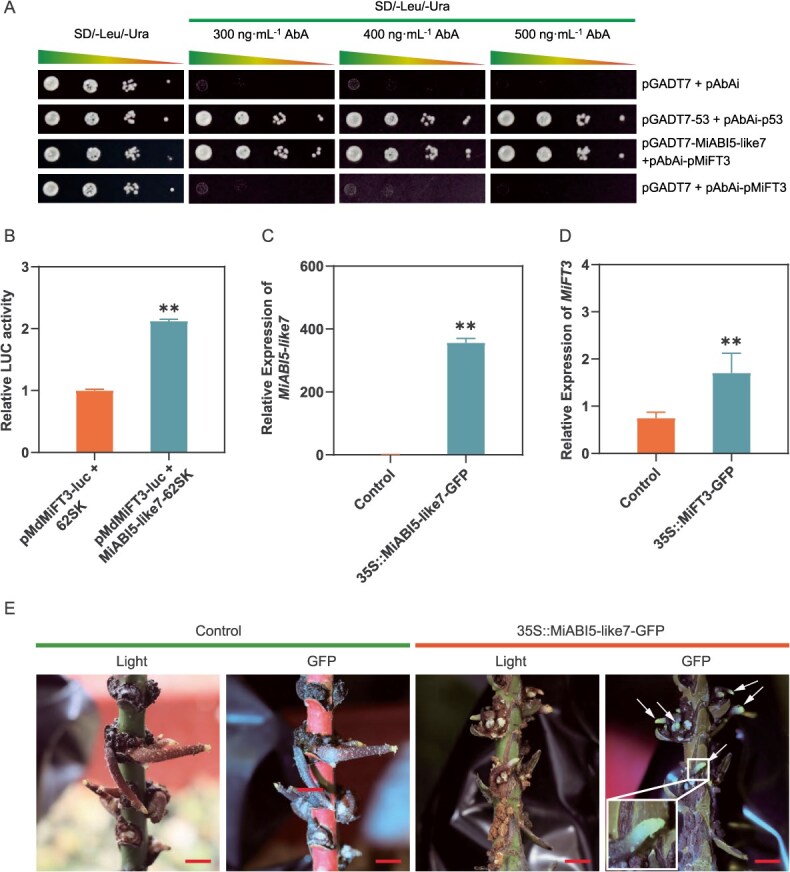
Transcript factor MiABI5-like7 directly activates the expression of *MiFT3.* (A) Y1H assay revealing binding of MiABI5-like7 to *MiFT3* promoter region. (B) LUC analysis validate the activation effect of MiABI5-like7 on the *MiFT3* promoter. An empty vector was transfected as the control. (C, D) Expression levels of *MiABI5-like7 and MiFT3* in MiABI5-like7-overexpressing mango hairy roots by RT-qPCR. Values are mean ± standard error. ^**^P < 0.01 (*n* = 3, Student's *t*-test). (E) MiABI5-like7-GFP-overexpressing hairy roots and GFP fluorescence from stems in mango. An empty vector without GFP was transfected as the control. Bars in E is 1 cm.

Given that the core flowering gene *MiFT3* is significantly induced by MC, we further investigated the regulatory mechanisms governing *MiFT3* expression. Utilizing the online tool PlantTFDB (http://planttfdb.gao-lab.org/blast.php), we analyzed the upstream transcription factors associated with *MiFT3* and identified several candidates, including bZIP transcription factors. Notably, *ABI5-like7*, a member of the bZIP family, exhibited strong co-expression with *MiFT3* during the MC-induced floral transition (Fig. 4E). Consequently, we hypothesize that ABI5-like7 serves as an upstream regulator of *MiFT3* in mango. To evaluate this hypothesis, we employed yeast one-hybrid (Y1H) assays to determine whether mango MiABI5-like7 can directly bind to the promoter of *MiFT3*. As illustrated in Fig. 6A, the results indicated that yeast cells harboring both MiABI5-like7 and MiFT3 constructs exhibited robust growth on selective media (SD/-Leu/-Ura) containing 300 to 500 ng·ml^−1^ Aureobasidin A (AbA), thereby confirming the interaction between MiABI5-like7 and the *MiFT3* promoter (Fig. 6A). This interaction was further elucidated through dual luciferase (LUC) analysis, which demonstrated that MiABI5-like7 significantly enhances the promoter activity of *MiFT3* (Fig. 6B). These findings suggest that MiABI5-like7 may directly activate the expression of *MiFT3* in response to MC.

To further elucidate the relationship between *MiABI5-like7* and *MiFT3* in mango, we conducted an experiment in which *MiABI5-like7* was overexpressed (35S::MiABI5-Like7-GFP) in mango hair roots, utilizing an empty construct as control (Fig. 6C). The overexpression of *MiABI5-like7* resulted in a significant upregulation of *MiFT3* expression in mango hair roots compared to the control hair roots (empty vector) under identical conditions (Fig. 6D and E), thereby demonstrating that MiABI5-like7 induces *MiFT3* expression in mango.

**Figure 7:**
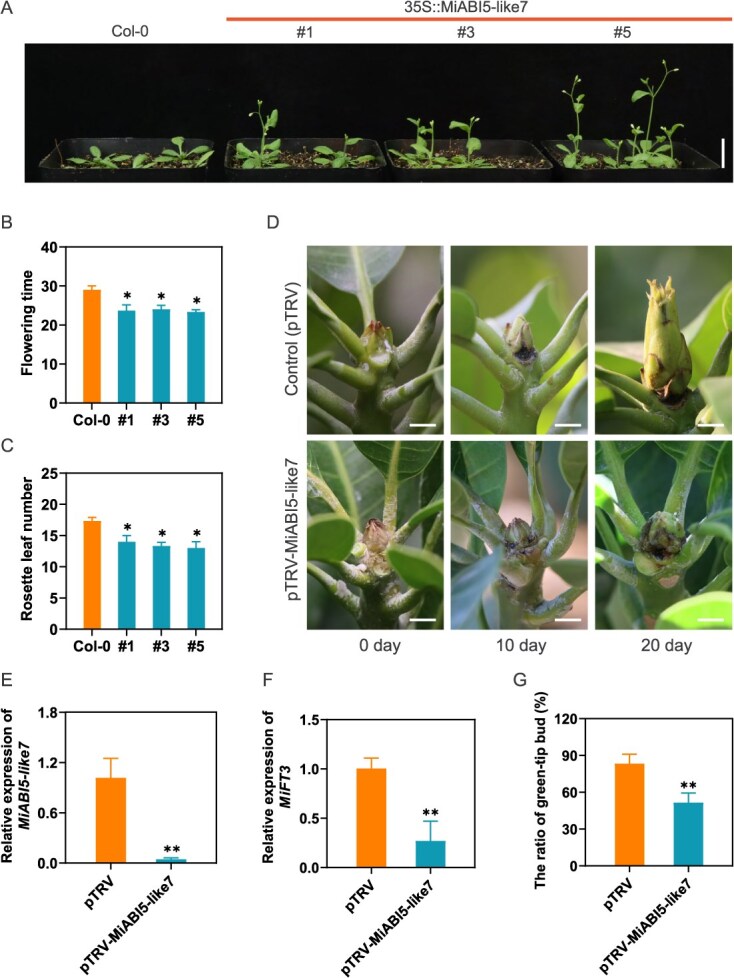
Functional analysis of *MiABI5-like7* in *Arabidopsis* and mango terminal bud. (A) Flowering phenotype of WT and 35S::MiABI5-like7-GFP transgenic plants. Bars in A is 1 cm. (B, C) Flowering time and rosette leaf number of 35S::MiABI5-like7-GFP transgenic *Arabidopsi*s lines. Values are mean ± standard error. ^*^P < 0.05 (Student's *t*-test). The flowering time and rosette leaf number were counted when plants bolted to 1 cm. (D) Developmental changes of mango buds in the control and *MiABI5-like7* silenced group. (E, F) *MiABI5-like7* and *MiFT3* expression levels in terminal buds at 10 days after VIGS treatment. (G) Silence of *MiABI5-like7* decreased the green-tip rate after 10 days of VIGS treatment. Values are mean ± standard error. ^**^P < 0.01 (*n* = 3, Student's *t*-test).

To investigate the role of MiABI5-like7 in the floral transition, we generated transgenic *Arabidopsis* plants overexpressing 35S::MiABI5-like7-GFP (Fig. 7A). Three independent transgenic *Arabidopsis thaliana* lines (OE-1, OE-3, and OE-5) were selected for phenotypic analysis of flowering. In comparison to the wild type (WT), all transgenic lines exhibited an early flowering phenotype and a significantly reduced number of rosette leaves under the same conditions (Fig. 7B and C), indicating that MiABI5-like7 contributes to the promotion of flowering.

To further elucidate the role of MiABI5-like7 in the floral transition of mango apical buds, a virus-induced gene silencing (VIGS) analysis was performed to silence *MiABI5-like7* in mango dormant buds of plants treated with MC for 70 DABT. After 10 days of infection treatment, the pTRV: MiABI5-like7 group inhibited the transition from vegetative bud to green-tip bud (Fig. 7D). The expression levels of *MiABI5-like7* and *MiFT3* in the pTRV: MiABI5-like7 group were significantly reduced compared to those in the blank pTRV treatment (Fig. 7E and F), leading to a notably lower green-tip ratio relative to the control group (Fig. 7G). Thus, we have confirmed that the expression of *MiABI5-like7* is crucial for floral transition in mango.

### MC-induced floral transition is dependent upon ABA signaling pathway

**Figure 8:**
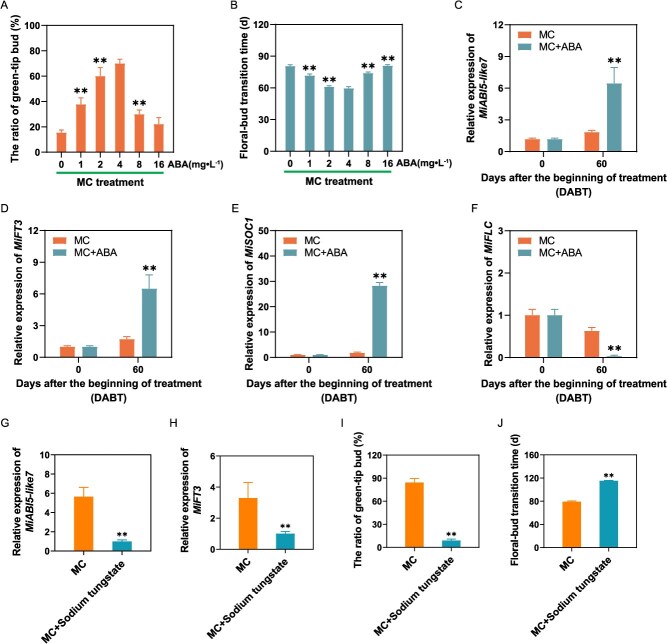
The role of ABA in MC-induced floral transition. (A) Statistics of the green-tip bud rate after incorporation of different concentrations of ABA into MC treatment. (B) Statistics of the floral–bud transition time after incorporation of different concentrations of ABA into MC treatments. The floral–bud transition time was counted when the green-tip ratio reached 50%. (C–F) *MiABI5-like7*, *MiFT3*, *MiSOC1*, and *MiFLC* expression levels after application of ABA (4 mg·l^−1^) + MC (2 g·l^−1^) and MC (2 g·l^−1^) alone. Values are mean ± standard error. **P < 0.01 (*n* = 3, Student's *t*-test). (G, H) *MiABI5-like7* and *MiFT3* expression levels after application of sodium tungstate (20 mM) + MC (2 g·l^−1^) and MC (2 g·l^−1^) alone. Values are mean ± standard error. ^**^P < 0.01 (*n* = 3, Student's *t*-test). (I) Statistics of the green-tip bud rate after spraying 20 mM sodium tungstate into MC-treated plants at 60DABT. (J) Statistics of the floral–bud transition time after spraying 20 mM sodium tungstate into MC-treated plants at 60DABT. Values are mean ± standard error. ^**^P < 0.01 (*n* = 3, Student's *t*-test).

To examine the role of ABA in floral transition induced by MC, varying concentrations of ABA were concurrently incorporated into MC (2 g·l^−1^) for the treatment of mango plants. The application of different ABA concentrations in conjunction with MC significantly influenced the timing of floral transition and the green-tip ratio (Fig. 8A and B). After 60 DABT, the green-tip bud ratio initially increased and subsequently decreased with escalating ABA concentrations (Fig. 8A). In comparison to MC treatment alone, the addition of 2 to 4 mg·l^−1^ ABA to MC markedly enhanced the green-tip bud ratio and significantly expedited the transition of flower buds (Fig. 8A and B). Moreover, the inclusion of ABA in MC notably up-regulated the expression of genes that positively regulate floral transition, such as *MiABI5-like7*, *MiFT3*, and *MiSOC1* (Fig. 7C–E), while it significantly down-regulated the expression of genes that negatively regulate flowering time, such as *MiFLC* (Fig. 8F). Furthermore, we applied sodium tungstate (20 mM), an ABA inhibitor, to mango plants treated with MC for 60 DABT. In comparison to the treatment with MC alone, the application of the ABA inhibitor markedly decreased the expression levels of *MiABI5-like7* and *MiFT3* (Fig. 8G and H), as well as the green-tip rate (Fig. 8I), consequently delaying the transition of flower buds by ~35 days (Fig. 8J). These findings suggest that the transition of mango flower buds induced by MC is reliant on the ABA signaling pathway.

## Discussion

### The application of MC promotes the process and efficiency of FI

In comparison to PBZ, MC is characterized as a plant growth retardant with a short half-life and a propensity for rapid degradation, and plays a significant role in the regulation of plant morphology, yield, and responses to abiotic stress [28, 30]. However, the influence of MC on FI in plants, particularly in woody fruit trees, remains inadequately explored. In this study, we observed that the green-tip bud ratio reached its peak by 80 DABT before subsequently declining in MC-treated group, whereas the control group exhibited a significantly lower ratio of green tip buds at various time points. Histological analysis revealed that at 80 DABT, the bud exhibited marked expansion in the lateral meristematic regions, representing the initiation of inflorescence primordium. In contrast, the bud of the control group predominantly displayed vegetative buds at all stages. These cytological changes align with previous research on the regulation of FI in mango by PBZ. During the 60 to 90 days of treatment with PBZ, the stem tip meristem underwent a morphological transition, losing its characteristic dome shape due to flattening, while exhibiting varying degrees of expansion in meristematic area [33]. However, the key time point of floral transition by PBZ treatment is vague. This study demonstrated that the 80 DABT was a critical phase for mango FI under the application of MC. Notably, the emergence of green-tip buds, characterized by the loosening of the apical bud and significant meristem expansion, serves as important indicators of the floral transition. Transcriptome analysis corroborated these findings, revealing that the number of commonly DEGs peaked at 80 days following MC treatment. Furthermore, KEGG enrichment analysis of these DEGs indicated significant enrichment of pathways related to plant hormone signaling and circadian rhythms during this period. The expression of the *MiFT3*, a key flowering integration factor, also reached its zenith at 80 DABT, with ratios in green tip buds exhibiting a consistent trend across different samples. The MC treatment resulted in a floral rate of 82.2%, significantly surpassing that of the control group. Consequently, the application of MC facilitated the expansion of mango apex bud cells and activated the genes associated with the plant hormone signal transduction pathway, thereby enhancing the process of mango FI and increasing the floral bud ratio.

### What is the molecular mechanism underlying MC in promoting mango floral transition?

During the FI process, floral transition determines the fate of the shoot meristem. It is evident that the application of exogenous MC can facilitate the floral transition and increase the flower bud ratio of mango. What molecular mechanism underlies this phenomenon? To investigate this issue, we conducted an in-depth analysis of the specific DEGs associated with floral transition induced by MC. Previous studies demonstrated that exogenous GA_3_ treatment inhibited the expression of the *FT* in woody fruit trees, including apple, citrus, mango, and loquat, suggesting that the *FT* plays a crucial role in regulating the flowering of woody plants in response to GA signaling [18–20, 34]. In this study, we employed WGCNA to identify a specific gene set associated with the floral transition in response to MC, comprising 1489 genes. This set includes several genes related to flowering, such as *MiFT3*, *MiSOC1-like*, and *MiCAL4*. The expression of these genes reached its peak 80 DABT with MC treatment, demonstrating a significant difference compared to the control group, thereby indicating their strong association with MC-induced floral transition. Within the co-expression network of flowering-related genes and TFs, the *MiFT3* (*Mi02g20180.1*) emerged as the pivotal gene. Three *MiFTs* (*MiFT1*, *MiFT2*, and *MiFT3*) have been identified in ‘SiJiMi’ mango, which exhibits multiple flowering cycles annually, overexpression of *MiFTs* promoted flowering under long-day conditions [32]. Nevertheless, our findings suggest that the *MiFT* (*Mi02g20180.1*), sharing 99% homology with the *MiFT3*, is the most crucial gene in response to MC-induced mango floral transition. Furthermore, the other two *MiFTs* were not identified in our transcriptomic data, which might be attributed to genetic differences among different varieties. Additionally, a caveat is that the co-expression network constructed from DEGs represents a trait-associated local network rather than a global regulatory network in this study, potentially excluding non–DEG-mediated pathways.

### The promotion of mango floral transition by MC is related to the GA pathway

Our transcriptomic analysis identified enrichment of the ‘plant hormone signal transduction’ and ‘diterpenoid biosynthesis’ pathways within the specific gene sets involved in flower transition induced by MC. This indicates that the GA biosynthesis and signal transduction pathways are closely related to the flower transition induced by MC. Exogenous application of GA_3_ contributed to GA homeostasis by inhibiting the expression of GA synthesis genes (*GA20ox* and *GA3ox*) and simultaneously promoting the expression of the GA catabolism gene *GA2ox* in *Paphiopedilum callosum*, thereby modulating GA concentration [4]. Conversely, GA biosynthesis inhibitors, such as PBZ and prohexadione-calcium, reduced GA content by up-regulating the expression of *GA20ox* and *GA3ox* while down-regulating *GA2ox* in crops such as tomatoes and strawberries [35, 36]. In this study, a notable feedback regulation of GA biosynthesis was observed 80 DABT with MC treatment, as evidenced by the significant upregulation of the *MiGA20oxs* and *MiGA3oxs* expressions. Concurrently, MC also induced the upregulation of three *MiGA2oxs* in mango. Interestingly, the Gh*GA2ox1*, *GhGA2ox3*, *GhGA2ox4,* and *GhGA2ox6* in cotton were significantly up-regulated under the induction of MC [30]. Therefore, we speculate that mango *MiGA2oxs*, similar to cotton, exhibits high sensitivity to MC and represents the crucial genes for MC-mediated inhibition of GA biosynthesis. Additionally, exogenous application of PBZ has been shown to promote mango flowering by reducing the levels of GA_4_, GA_3_, GA_7_, and GA_1_ in the buds [5]. However, our findings further demonstrate that MC treatment significantly decreases the levels of GA_20_ and GA_3_ in mango buds, suggesting that MC primarily influences the GA_13_-hydroxylation pathway in GA biosynthesis.

Beyond the activity of GAs, our research also indicates that DELLAs play a role in the floral transition induced by MC. Among the six identified DELLAs, four genes exhibited significant upregulation, while two genes demonstrated significant downregulation at 80 DABT with MC treatment. This observation is consistent with previous findings, such as PBZ significantly up-regulated DELLAs expression levels in sorghum [37], and prohexadione-calcium significantly down-regulated DELLAs expression levels in strawberries [36]. A recent study identified two *MiDELLAs*, *MiSLR1* and *MiSLR1*, and demonstrated that PBZ and prohexadione-calcium markedly decreased the expression of *MiSLR1* while increasing that of *MiSLR2* in mango [38]. Subsequent overexpression assays in *Arabidopsis thaliana*, along with protein interaction analyses, further suggested that MiSLR1 and MiSLR2 play integral roles in the regulation of mango flowering. It has also confirmed the presence of antagonistic interactions among DELLA proteins. For instance, SCL3 has been shown to antagonize other DELLA proteins to maintain GA homeostasis [39]. In *Arabidopsis thaliana*, a decrease in GA levels within the root elongation zone resulted in the accumulation of DELLA proteins, which subsequently inhibit DELLA RNA levels [40]. Furthermore, DELLA proteins have been demonstrated to interact directly with *ABI4* and *ABI5*, playing a role in the seed germination process of *Arabidopsis thaliana* [41, 42]. A recent study also identified that the regulatory modules of DELLA proteins RGL2 and ABI4 mediate the antagonistic effects between ABA and GA [43]. Consequently, we hypothesize that the floral transition induced by MC may involve an antagonistic interaction between the up-regulation and down-regulation of DELLAs expression. This interaction appears to serve a dual purpose: maintaining GA homeostasis and regulating mango floral transition through interactions with other hormonal pathways, such as the ABA signal transduction pathway. Nonetheless, the precise functions and mechanisms of action of these DELLAs necessitate further comprehensive investigation.

### The promotion of mango floral transition by MC depends on the ABA pathway

Throughout various plant developmental processes, GA often exhibit antagonistic interactions with ABA. For instance, GA facilitate seed germination, whereas ABA acts as an inhibitor [44]. Nevertheless, ABA significantly contributes to the process of FI in some woody plants. Specifically, PBZ treatment has been shown to significantly elevate ABA levels in mango buds, thereby promoting flower evocation [4]. Similarly, exogenous ABA application has been found to enhance flower bud formation in apple and lychee, while ABA inhibitors have been observed to suppress flowering [22, 23]. In this study, MC treatment markedly increased the expression of the ABA biosynthesis gene *MiNCED1* and elevated endogenous ABA levels, thereby inducing floral transition. These findings suggest that the antagonistic interaction between GA and ABA is closely related to the floral transition of perennial woody plants. Nevertheless, the precise mechanisms by which the ABA signaling pathway responds to GA signals and participates in floral transition remain inadequately understood.

Notably, ABI5, a pivotal effector in ABA signaling, plays an essential role in hormone crosstalk [45]. In this study, MC was found to induce the expression of *MiABI5-like7*, thereby facilitating floral transition. Heterologous overexpression of *MiABI5-like7* in *Arabidopsis thaliana* resulted in the phenotype of early flowering. Conversely, silencing *MiABI5-like7* in the terminal buds of mango led to a reduction in the expression of *MiFT3*, delayed the transition of flower bud, and significantly decreased the green-tip bud rate. These findings clearly demonstrate that *MiABI5-like7* serves as a crucial regulatory gene in mango floral transition induced by MC. Furthermore, we have confirmed for the first time the direct binding interaction between MiABI5-like7 and the *MiFT3* promoter using a mango hair-like root transgenic system, in conjunction with yeast one-hybrid and dual-luciferase assays. Collectively, these results suggest that MC induces the expression of flowering genes via ABA signaling, thereby promoting floral transition in mango. Exogenous application of ABA has been shown to promote early flowering in mango induced by MC, primarily through the upregulation of *MiABI5-like7* expression. Conversely, ABA inhibitors significantly prolong the time for MC-induced flower bud formation by downregulating *MiABI5-like7* expression. These findings suggest that MC-induced floral transition is dependent on ABA. Previous studies have demonstrated that ABI5 and ABI4 delays flowering in *Arabidopsis thaliana* by activating the expression of the flowering inhibitory gene *FLC* [24]. Recent research in rice has revealed that ABA inhibits flowering through the SAPK8-ABF1-Ehd1/Ehd2 pathway [21]. In summary, this study elucidates a novel regulatory pathway for flowering in perennial woody fruit trees, wherein floral transition is induced by ABA signals. While the mechanisms of ABA action differ across species, the findings of this research, though preliminary, offer significant insights into the intricate role of ABA in the regulation of plant flowering.

In summary, our research elucidated the pivotal role of MC in the floral transition of mango, emphasizing that this MC-induced transition is contingent upon the ABA signaling pathway. In the absence of exogenous MC, mango buds exhibit low expression levels of GA degradation genes (*MiGA2oxs*), resulting in elevated concentrations of GAs (GA_20_ and GA_3_). Concurrently, there is a low expression of ABA synthesis genes (*MiNCED1*) and a high expression of ABA degradation genes (*MiCYP707A1s*), culminating in reduced ABA content within the buds. The elevated GA levels coupled with diminished ABA levels inhibit the activation of the MiABI5-like7-MiFT3 module-mediated pathway, which is crucial for floral transition, thereby leading to a reduced rate of floral bud. Conversely, the application of exogenous MC decreases endogenous GA_20_ and GA_3_ levels in the buds by up-regulating the expression of GA degradation genes (*MiGA2oxs*) and enhances ABA content by up-regulating ABA synthesis genes (*MiNCED1*) while down-regulating ABA degradation genes (*MiCYP707A1s*). This modulation results in altered expression levels of GA signaling pathway genes (*MiDELLAs*) and activation of the ABA signaling pathway gene MiABI5-like7. The MiABI5-like7 transcription factor interacts with the *MiFT3* promoter to up-regulate *MiFT3* expression, thereby facilitating floral transition and increasing the floral bud rate. This research introduces a novel inducer aimed at enhancing floral transition in perennial tropical fruit trees within warm climates. It offers a potential solution to the challenge of inadequate flowering during mild winters and supports the development of cultivars with robust flowering capabilities.

## Materials and methods

### Plant materials and growth conditions

The mango cultivar used in this study was ‘Tainong No. 1’, the most widely cultivated variety in the coastal areas of South China, which was grown in the experimental orchard of South Subtropical Crops Research Institute, Zhanjiang, China (110° 16′ E, 21° 10′ N) under a subtropical climate with the average temperature of about 23°C and an occasional temperature drop below 15°C overnight in December. Fifteen-year-old ‘Tainong No. 1’ mango trees grafted on ‘Yuexi No. 1’ rootstocks were managed conventionally during the growth period, including pruning, fertilization and disease and pest control from 2020 to 2024.

### Treatment and sample preparation

Mango trees were selected randomly at flush growth cessation stage when terminal buds were of transient dormancy in late September. On 25 September 2020, the whole plants were treated with 2 g·l^−1^ of MC (Yuanye, S18143), applied every 10 days for a total of three applications, with a water spray as the control. Each treatment group consisted of three biological replicates, with each replicate comprising 20 trees. Buds were collected at 0, 60, 80, and 100 DABT, and then were immediately frozen in liquid nitrogen and stored at −80°C for subsequent transcriptomic and phytohormonal analyses.

On 28 September 2023, plants were sprayed with a mixture of 2 g·l^−1^ MC and varying concentrations of ABA (Yuanye, S18005) (1, 2, 4, 8, and 16 mg·l^−1^), applied at 10-day intervals for a total of three applications, with MC alone serving as the control. At 60 DABT, buds were collected for gene expression analysis.

On 26 September 2024, plants were treated with 2 g·l^−1^ MC at 10-day intervals for three applications. At 60 DABT, plants were additionally treated with 20 mM sodium tungstate (Yuanye, S61169) [22], with MC alone as the control. Buds were collected at 80 DABT for gene expression analysis.

### Determination of ratios of new shoot and floral bud

A statistical analysis of the new shoot ratio under natural conditions was conducted annually between October and November from 2020 to 2022. The experiment was designed with three biological replicates, with each replicate comprising three trees. For each tree, 30 branches were marked and continuously monitored to record new shoot formation. Ratio of new shoot (%) = (Number of branches bearing new shoots / 30) × 100. Furthermore, the floral bud ratio under natural conditions was assessed during the flowering period. The assessment included three biological replicates, with each replicate comprising three trees. For each tree, 30 buds were marked for analysis. Ratio of floral bud (%) = (Number of floral bud / 30) × 100.

### Determination of ratios of green-tip and initial-floral bud and the transition time of floral bud

In 2020, ~30 buds of each tree on different treatments were labeled, and the ratios of green-tip and initial-floral bud were measured. The formula used to calculate the percentage of green-tip or initial-floral bud was as follows: Ratio of green-tip or initial-floral bud (%) = (Number of green-tip or initial-floral bud / 30) × 100. In 2023, the green-tip bud ratio was recorded from 50 DABT, and the transition time for floral bud was documented. In 2024, the green-tip bud ratio was recorded from 80 DABT, and the transition time for floral bud was documented.

### Histological observation

The bud samples were fixed in FAA solution (70% ethanol:37% formaldehyde:glacial acetic acid = 18:1:1) and stored at 4°C after vacuumizing. Detailed steps of sample preparation and paraffin sectioning refer to the previously described method [46].

### RNA-seq

Twenty-four cDNA libraries were constructed and sequenced using the Illumina HiSeq 2000 platform, generating 150-bp paired-end reads. The raw sequencing data were processed using fastp version 0.19.3 to obtain high-quality reads, which were subsequently aligned to the mango reference genome [47] employing HISAT version 2.1.0. Gene alignment was quantified using featureCounts version 1.6.2, followed by the calculation of FPKM (Fragments Per Kilobase of transcript per Million mapped reads) for each gene, taking into account gene length. Differential expression analysis between the two groups was conducted using DESeq2 version 1.22.1, with *P* values adjusted via the Benjamini & Hochberg method to control for false discovery rate. DEGs were identified using threshold values of fold-change ≥2 and FDR < 0.05. Additionally, PCA and Pearson's correlation coefficient were employed to evaluate inter-group differences and the consistency of sample replicates within groups. Furthermore, Venn diagrams and gene expression patterns were generated using TBtools [48].

### WGCNA and gene set enrichment

To identify co-expression modules highly relevant to MC-induced flowering, DEGs were identified through pairwise comparisons following the previously methodology outlined [49, 50]: (i) each stage of the MC treatment group vs the preceding stage; (ii) the same stage of the treatment group vs the control group. Duplicate genes were removed, resulting in 4847 DEGs. The average FPKM values of 4847 genes were utilized in the WGCNA package in R, and the gene connections within the network adhered to a scale-free network distribution [51]. The parameters used for WGCNA construction were set as follows: a soft-thresholding power of 14, a minimum module size of 30, and a merging height threshold of 0.25. The phenotypes are 8 biologically defined stage-treatment combinations (At, Bt, Ct, Dt, TAt, TBt, TCt, TDt), with each stage-treatment combination as a phenotype to identify modules associated with stage or MC-specific responses. Binary coding (1 for the target group, 0 for others; Table S7) was used to quantify associations [49]. The eigenvalue of each module was computed and employed to assess the association with each coded trait. By calculating the gene connectivity kME based on the feature genes, 9 stage- or MC-specific modules were identified. The network visualization was performed using Cytoscape (version 3.9.1).

A total of 1489 genes from the MEgreen module were subjected to KEGG enrichment analysis using the Metware Cloud Platform (https://cloud.metware.cn). From this analyzed gene set, genes were further selected for subsequent investigation based on their relevance to MC-induced flowering—specifically those annotated to the ‘Plant hormone signal transduction’, ‘Diterpenoid biosynthesis’, and ‘Carotenoid biosynthesis’ pathways (Table S8).

### Determination of ABA and GAs concentrations

The concentrations of ABA and GAs in the bud of mango were quantified following the protocol of Šimura *et al*. [52]. The analysis of ABA and GAs were determined using a liquid chromatography-electrospray ionization-tandem mass spectrometry (LC-ESI-MS/MS) system.

### Y1H assay

The Matchmaker Gold Yeast One-Hybrid System Kit (Clontech) was employed for Y1H analysis. Initially, the *MiFT3* promoter sequence was ligated into the pABAi vector, while the CDS of *MiABI5-like7* was inserted into the pGADT7 vector. The linearized pABAi-proMiFT3 vector was subsequently transformed into Y1H Gold yeast strain cells and cultured on SD medium lacking uracil (SD/-Ura). These positive clones were then plated on SD/-Ura medium supplemented with varying concentrations of AbA to assess self-activation and determine the optimal AbA concentration. Subsequently, the construct containing MiABI5-like7 was introduced into the yeast strain harboring pABAi-proMiFT3. Following confirmation of positive transformation, the yeast cultures were plated on medium containing AbA to monitor yeast growth. Primers are detailed in Table S6.

### LUC assay

The *MiFT3* promoter sequence was ligated to the pGreenII 0800-LUC vector, and the CDS sequence of *MiABI5-like7* was inserted into the pGreenII-62-SK vector under the control of the 35S promoter. Following the transformation of GV3101 with these constructs, the transformed agrobacteria were combined in a 4:1 volume ratio and used to infect tobacco leaves. Three experimental replicates were conducted, utilizing empty vectors as controls. Tobacco leaves were harvested after 36 to 48 hours of incubation. The Firefly Luciferase Reporter Assay System (Promega Corp., Madison, WI, USA) was employed to quantify firefly luciferase (LUC) and sea kidney luciferase (REN) activities [53]. Primers are detailed in Table S6.

### Overexpression assays in mango hair roots and *Arabidopsis thaliana*

The CDS of *MiABI5-like7* was inserted into the pCAMBIA1300-GFP vector, resulting in the construction of the 35S::MiABI5-like7-GFP expression vector. The 35S::MiABI5-like7-GFP expression vector was introduced into the hair root strain MSU440 for the transformation of mango roots and *Arabidopsis thaliana*. Primers are detailed in Table S6.

The mango hair root transformation system was optimized based on the previously described method [54]. Initially, single colonies containing the recombinant plasmids were selected and cultured until an OD600 of 0.8 was reached. Subsequently, the bacterial cells were collected and re-suspended in a buffer solution containing 10 mM MES, 10 mM MgCl_2_, and 100 μM acetosyringone, and incubated for 2 hours to prepare the infecting bacterial solution. In this study, 18- to 20-day-old genetically uniform ‘Tainong No. 1’ seedlings, derived from nucellar embryos, were chosen as the transformation subjects. Incisions were made on the seedling stems, which were then wrapped with fat-free cotton soaked in the bacterial solution. MSU440 carrying an empty vector served as the control. After a 12-hour incubation period, the wound was covered with black plastic film. The substrate for seedling cultivation was regularly watered, and the growth environment was controlled at 26°C to 28°C with a 16-hour light/8-hour dark photoperiod. Following 45 days of cultivation, the presence of GFP was detected using a dual-wavelength fluorescent protein excitation lamp (LUYOR-3415). Each experiment included three biological replicates, with each replicate consisting of five independent nucellar seedlings and three technical replicates for molecular assays (e.g. RT-qPCR).

The MiABI5-like7 transgenic *Arabidopsis* plants were generated through *Agrobacterium tumefaciens* GV3101-mediated floral dip transformation [55]. The transgenic seeds were subsequently selected on MS medium supplemented with 50 mg·l^−1^ hygromycin. Homozygous T3 transgenic lines were established for phenotypic characterization, focusing on parameters, such as flowering time and rosette leaf count.

### VIGS assay

The mango bud VIGS assay was refined in accordance with the previous method [56–58]. Initially, the 350-bp CDS fragment of *MiABI5-like7* was inserted into the vector pTRV2 to construct the recombinant virus vector pTRV2-MiABI5-like7. Following sequence verification, plasmids pTRV2-MiABI5-like7, pTRV1, and pTRV2 were individually transformed into GV3101. Subsequently, an *Agrobacterium* solution containing pTRV2-MiABI5-like7 and pTRV1, mixed in a 1:1 ratio, was utilized to infect mango buds of plants treated with MC for 70 DABT, while an *Agrobacterium* solution containing pTRV2 and pTRV1 (1:1) served as the negative control. The infection procedure was as follows: a 1-ml syringe was used to inject the bacterial suspension into the base of bud, specifically at the juncture between the bud and the branch. The injection site and the entire bud were then covered with a degreased cotton ball saturated with the same agrobacterial solution; this covering was maintained for 48 hours to retain moisture and ensure prolonged contact between the agrobacteria and bud tissues. The experimental design included three replicates, each comprising 30 dormant buds; 10 buds were allocated for analyzing silencing effects, and 20 buds were used to calculate the ratio of green-tip bud. Samples were collected 10 days post-infection, and RT-qPCR was employed to assess gene silencing in the terminal bud, while the proportion of green-tip bud was determined.

### RT-qPCR analysis

Total RNA was extracted from buds using the EASYspin Rapid Plant RNA Extraction Kit (Aidlab Biotech, Beijing). Reverse transcription was conducted with HiScript® Q Select RT SuperMix for qPCR Kit (Nanjing Nuoweizan Company, Catalog No.: R312-02) following the manufacturer's protocol. The RT-qPCR reactions were performed using the DyNAmo Flash SYBR Green qPCR Kit (Thermo, USA), adhering to the specified experimental procedures. The following conditions were applied: pre-incubation at 95°C for 5 minutes, followed by 40 cycles of 95°C for 10 seconds, 60°C for 20 seconds, and 72°C for 10 seconds. Gene expression levels were calculated using the formula 2^−ΔΔT^. Quantitative analysis of the target genes was conducted using the LightCycler480 II system (Roche, Switzerland). The primer sequences are detailed in Table S6.

### Statistical analysis

One-way ANOVA and student’s *t*-test were used on GraphPad Prism version 8.0 (https://www.graphpad.com) to analyze significant differences, which was indicated by asterisks in the figures.

## Supplementary Material

Web_Material_uhaf336

## Data Availability

All data included in the study were publicly available. All experiment data are provided in the attachment. The raw RNA-seq data are accessible at NCBI under the BioProject PRJNA1295084.
