# Supplementary figures and images for: MiABI5-like7-MiFT3 regulatory module controls floral transition induced by mepiquat chloride in evergreen perennial mango (Mangifera indica L.)
Source: Hortic Res. 2025 Dec 8;13(3):uhaf336. doi: 10.1093/hr/uhaf336 (PMC13035463; doi:10.1093/hr/uhaf336)

a

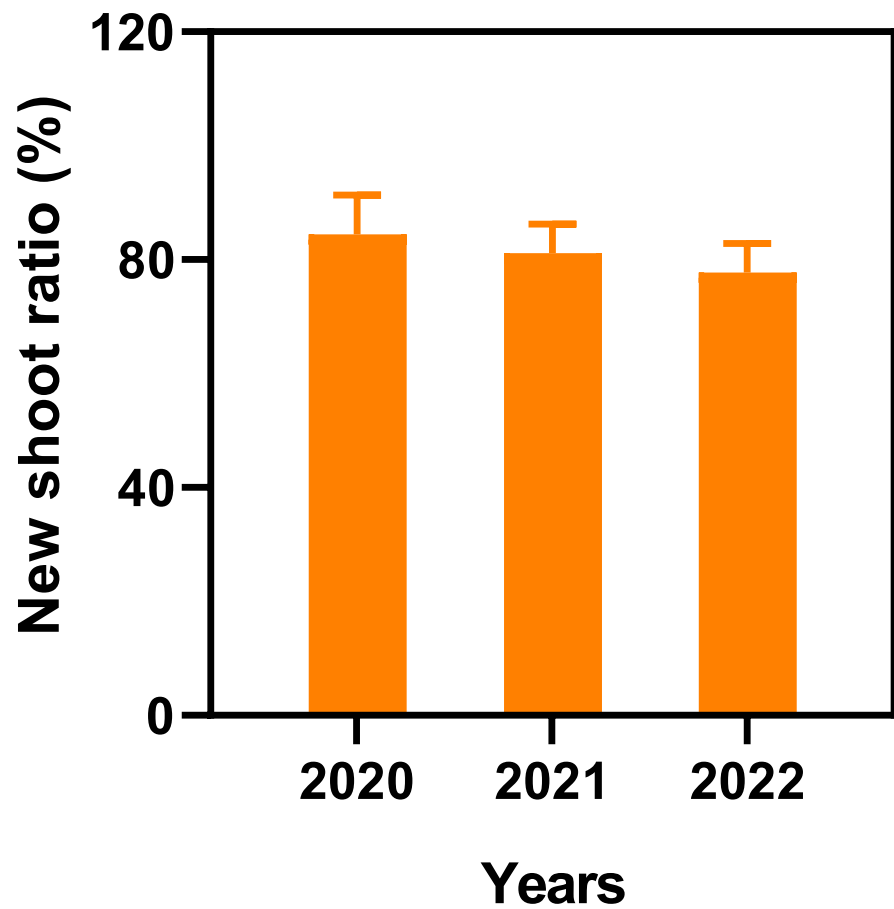

b

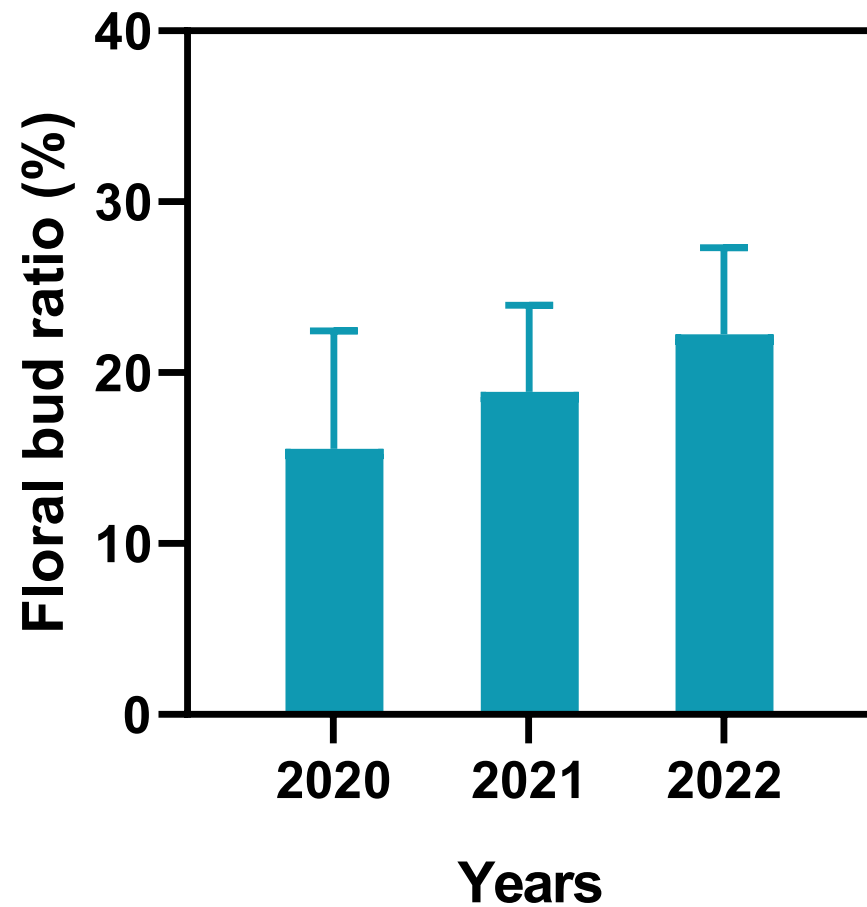

Supplement: Web_Material_uhaf336 [file web_material_uhaf336.zip › Figure S1.pdf]

a

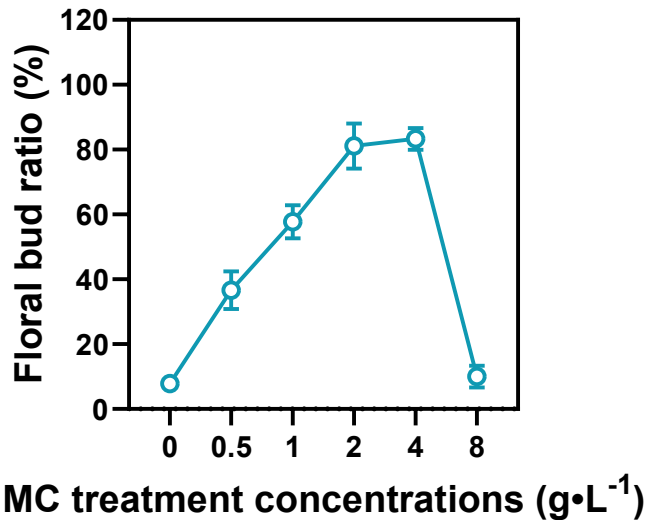

b

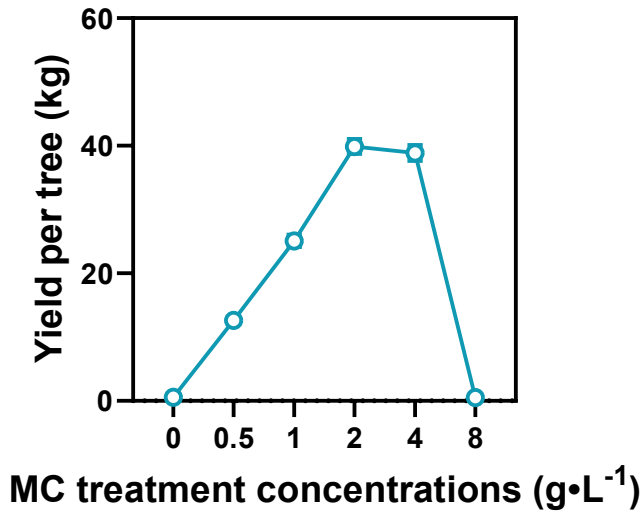

Supplement: Web_Material_uhaf336 [file web_material_uhaf336.zip › Figure S2.pdf]

a

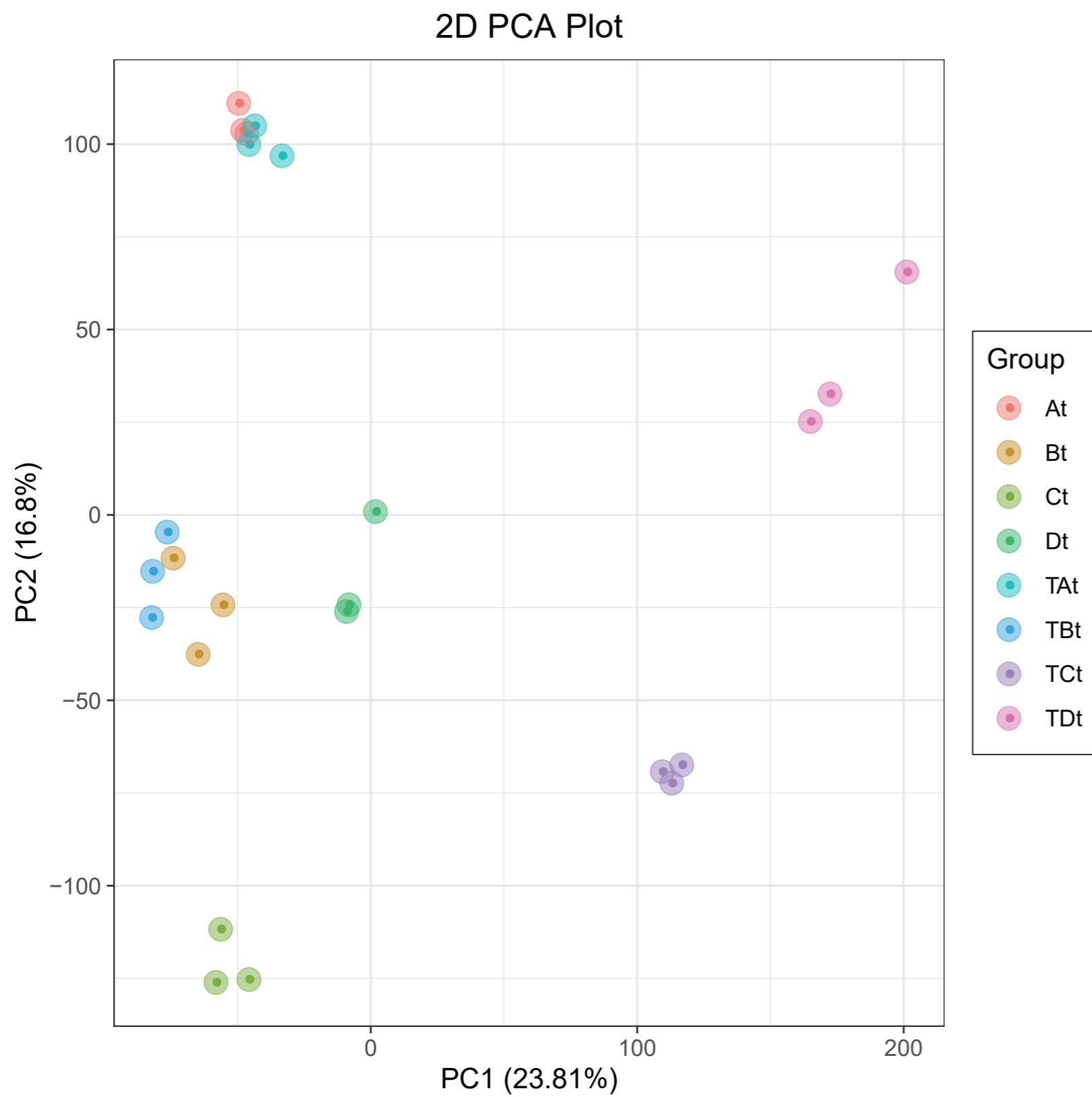

b

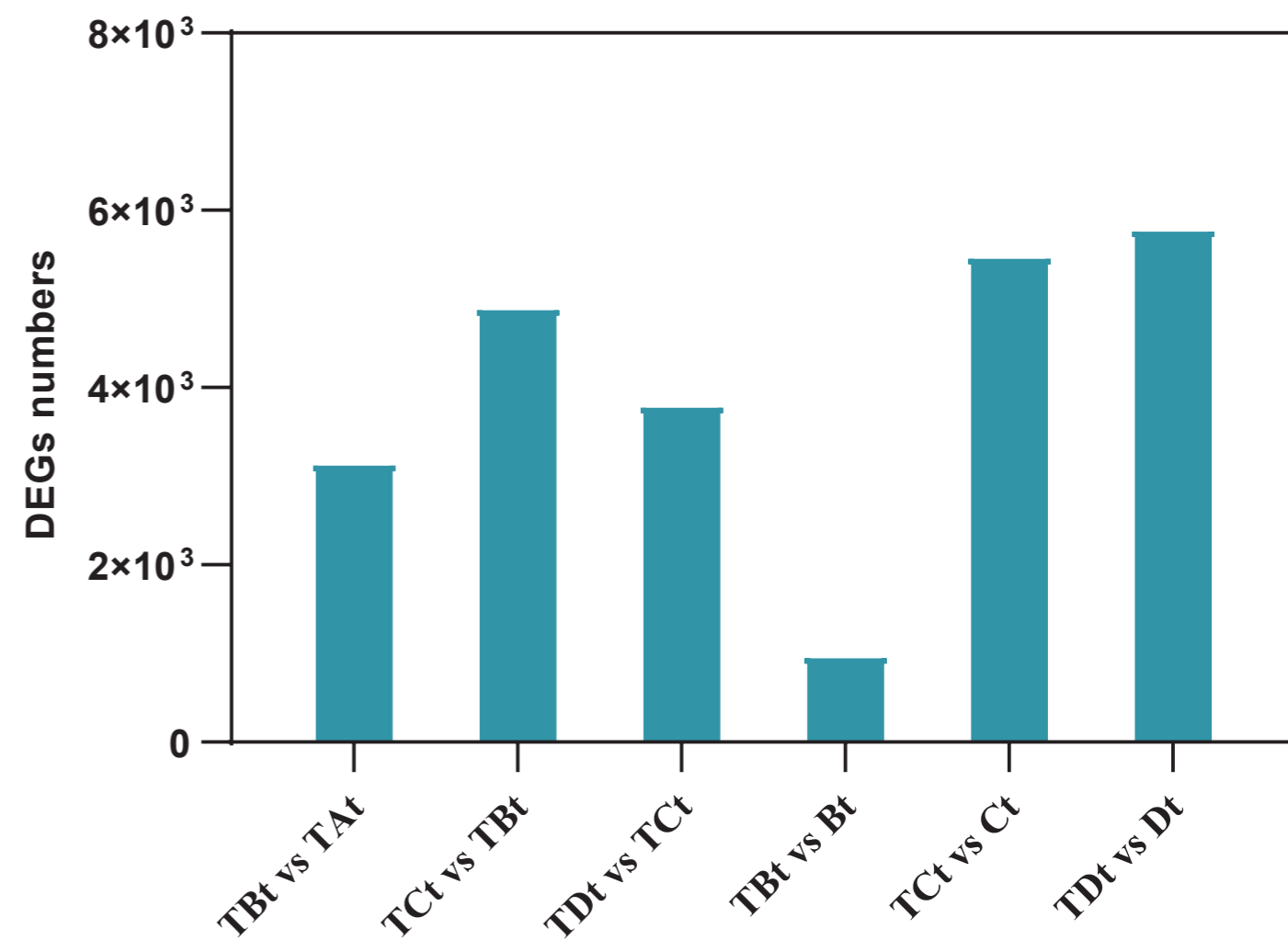

Supplement: Web_Material_uhaf336 [file web_material_uhaf336.zip › Figure S3.pdf]

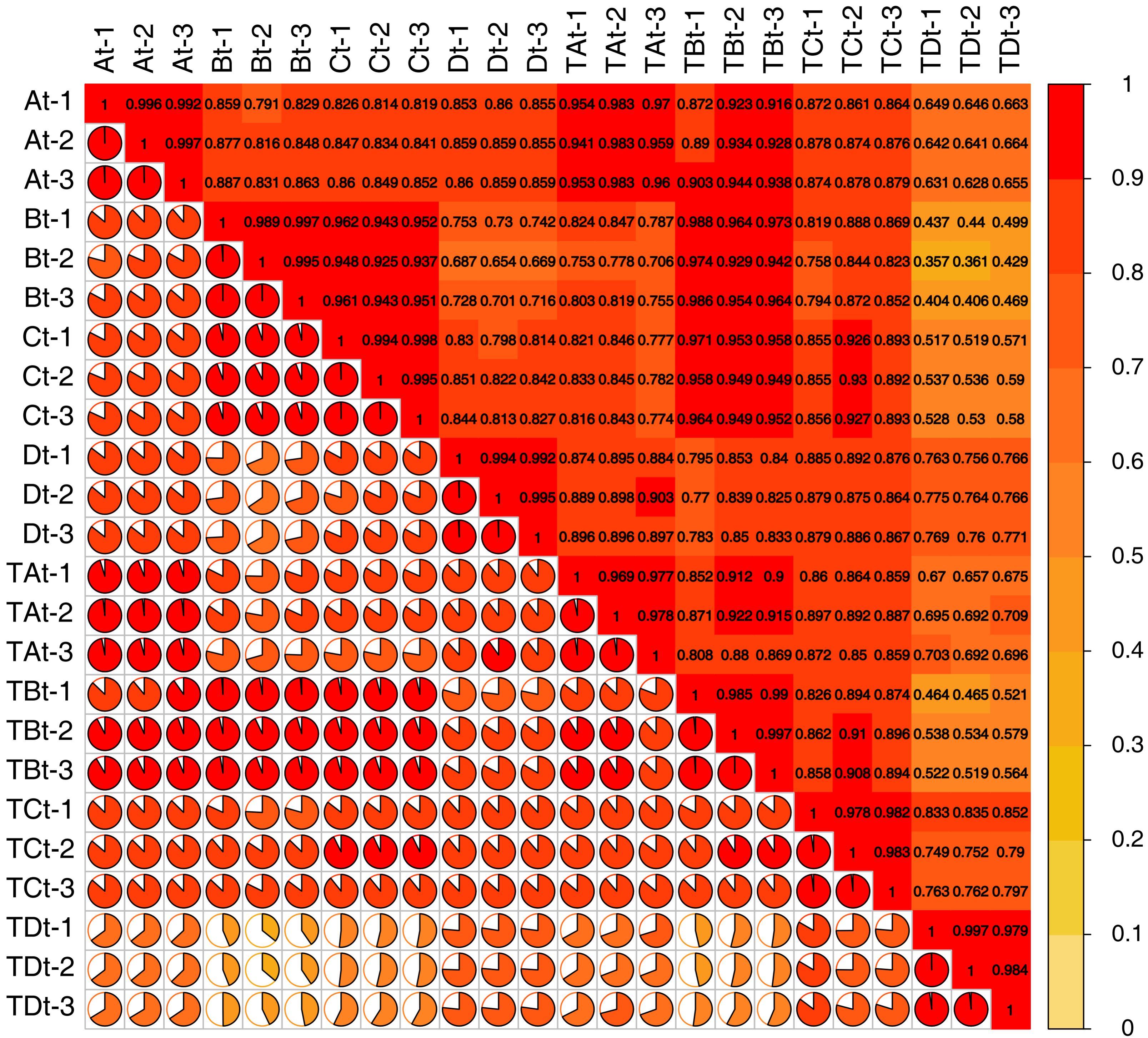

Supplement: Web_Material_uhaf336 [file web_material_uhaf336.zip › Figure S4.pdf]

a

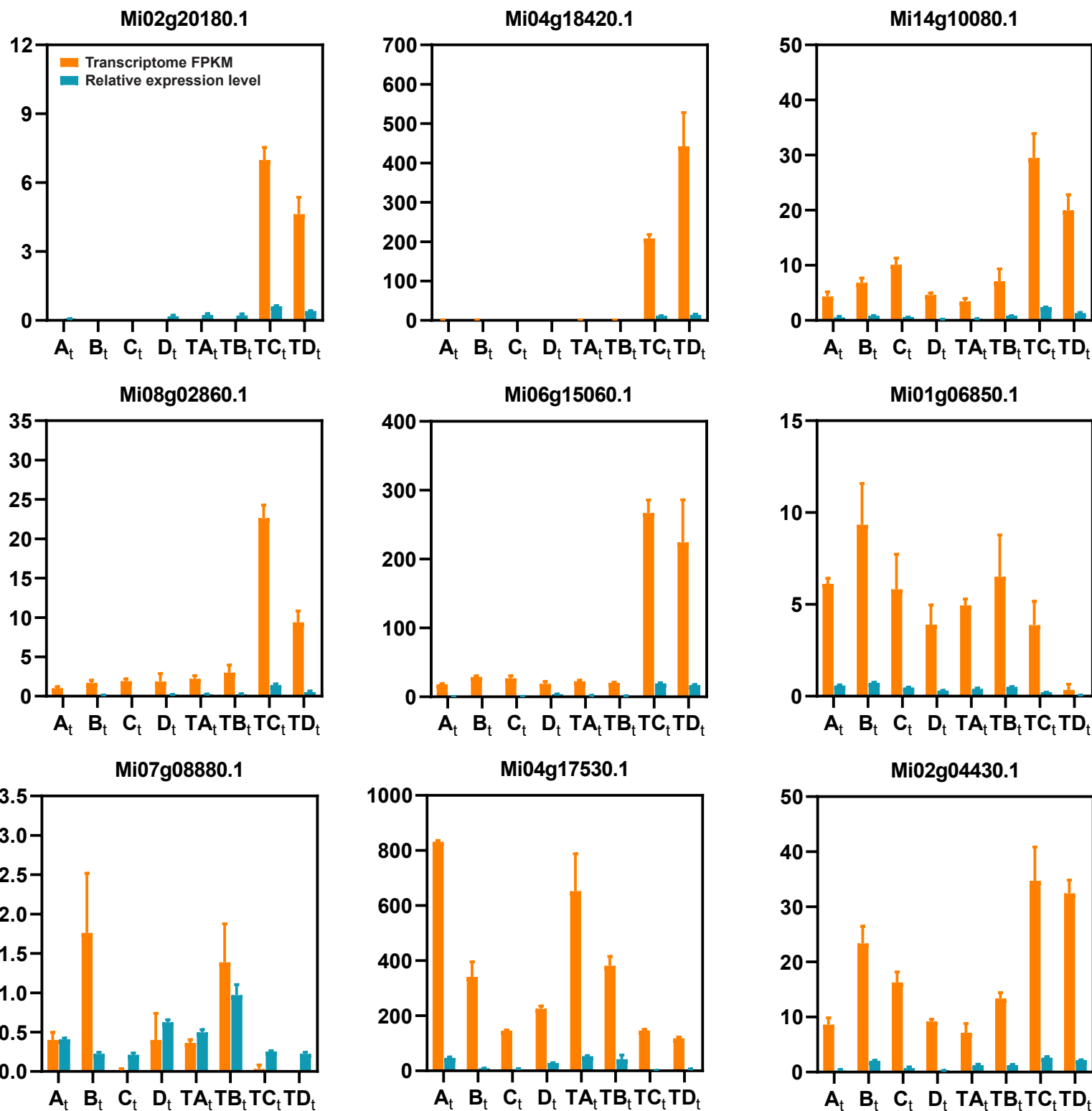

b

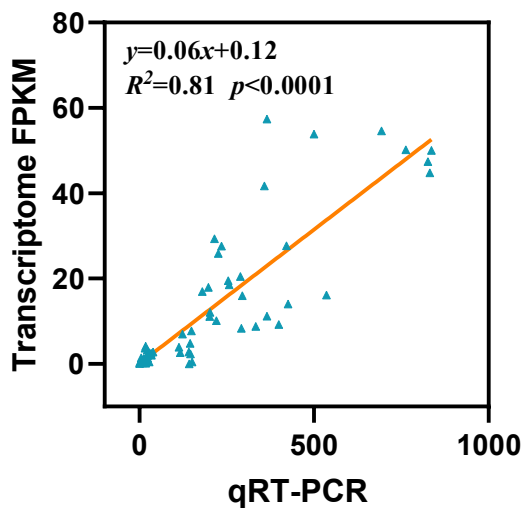

Supplement: Web_Material_uhaf336 [file web_material_uhaf336.zip › Figure S5.pdf]
